# Syphilis and Some Affections Which Resemble It

**Published:** 1899-02

**Authors:** 


### SYPHILIS AND SOME AFFECTIONS WHICH RESEMBLE IT.

At the West Point meeting of the American Association of Genito-urinary Surgeons, Dr. Fordyce read a paper on this subject, says the *Charlotte Medical Journal.* He stated that while, as a rule, syphilis reveals itself by clearly defined features and symptoms, cases are not infrequently met with which demand all the experience and knowledge of the expert to determine their true nature. It may be truthfully stated that no other condition, with the possible exception of tuberculosis, presents such widely diversified phenomena and such important relationship with both internal medicine and surgery. We can not always appeal with success to the microscope to settle doubtful cases, even where the lesions are accessible to excision. We have, fortunately, in the therapeutic test a potent means of determining the nature of certain doubtful cases; but even here there are possibilities of error which occasionally confront us. While it is frequently possible to make a diagnosis of syphilis with the appearance of the initial sore, experience has taught us to be conservative in this respect, and generally advise the patient to await the development of positive signs of constitutional infection before beginning the use of mercury.

Error is more apt to arise in the diagnosis of extragenital chancres, for the possibility of primary syphilis away from the genital organs is not always borne in mind by the practitioner. The doctor said he personally knew of several instances in which the primary sore of the lip and face was excised under the mistaken diagnosis of epithelioma, the subsequent development of constitutional symptoms rendering the character of the disease unmistakable.

In the diagnosis of the secondary stage of syphilis, the acute exanthemata, drug eruptions, and a multitude of non-venereal eruptions may confuse the physician if all the concomitant symptoms are not given proper consideration. He recalled one instance where a patient with a smallpox eruption was admitted to one of our large city hospitals with the erroneous diagnosis of syphilis. The mistake was not discovered until the death of the patient and the outbreak of variola among a number of exposed patients and hospital attendants.

The iodides in susceptible individuals sometimes produce pustular and ulcerative eruptions which simulate very closely the rupial lesions in syphilis, and, as the iodides are employed to combat such an eruption, it is sometimes difficult to distinguish the one from the other.

In the so-called tertiary stage of syphilis the disease is again prone to localize itself, and can, in the skin, imitate tuberculous processes or malignant disease; in the subcutaneous tissues, new growths of malignant nature; in bone, tuberculosis or sarcoma; in the testes, tuberculosis or other neoplasms. Syphilis of the tongue is one of the most common antecedents of cancer of this organ, it being sometimes difficult to determine when syphilis ceases and when epithelioma begins.—*Practical Medicine.*

